# Microbial Metabolism and Community Dynamics in Hydraulic Fracturing Fluids Recovered From Deep Hydrocarbon-Rich Shale

**DOI:** 10.3389/fmicb.2019.00376

**Published:** 2019-03-12

**Authors:** Yuki Morono, Jessie R. Wishart, Motoo Ito, Akira Ijiri, Tatsuhiko Hoshino, Marta Torres, Circe Verba, Takeshi Terada, Fumio Inagaki, Frederick S. Colwell

**Affiliations:** ^1^Kochi Institute for Core Sample Research, Japan Agency for Marine-Earth Science and Technology, Kochi, Japan; ^2^Research and Development Center for Submarine Resources, Japan Agency for Marine-Earth Science and Technology, Yokosuka, Japan; ^3^National Energy Technology Laboratory, United States Department of Energy, Albany, OR, United States; ^4^College of Earth, Ocean, and Atmospheric Sciences, Oregon State University, Corvallis, OR, United States; ^5^Marine Works Japan, Yokosuka, Japan; ^6^Research and Development Center for Ocean Drilling Science, Japan Agency for Marine-Earth Science and Technology, Yokohama, Japan

**Keywords:** NanoSIMS, hydraulic fracturing, microbial metabolism, microbial community, N and C assimilation

## Abstract

Hydraulic fracturing is a prominent method of natural gas production that uses injected, high-pressure fluids to fracture low permeability, hydrocarbon rich strata such as shale. Upon completion of a well, the fluid returns to the surface (produced water) and contains natural gas, subsurface constituents, and microorganisms (Barbot et al., 2013; Daly et al., 2016). While the microbial community of the produced fluids has been studied in multiple gas wells, the activity of these microorganisms and their relation to biogeochemical activity is not well understood. In this experiment, we supplemented produced fluid with ^13^C-labeled carbon sources (glucose, acetate, bicarbonate, methanol, or methane), and ^15^N-labeled ammonium chloride in order to isotopically trace microbial activity over multiple day in anoxic incubations. Nanoscale secondary ion mass spectrometry (NanoSIMS) was used to generate isotopic images of ^13^C and ^15^N incorporation in individual cells, while isotope ratio monitoring–gas chromatography–mass spectrometry (IRM–GC–MS) was used to measure ^13^CO_2_, and ^13^CH_4_ as metabolic byproducts. Glucose, acetate, and methanol were all assimilated by microorganisms under anoxic conditions. ^13^CO_2_ production was only observed with glucose as a substrate indicating that catabolic activity was limited to this condition. The microbial communities observed at 0, 19, and 32 days of incubation did not vary between different carbon sources, were low in diversity, and composed primarily of the class *Clostridia*. The primary genera detected in the incubations, *Halanaerobium* and *Fusibacter*, are known to be adapted to harsh physical and chemical conditions consistent with those that occur in the hydrofracturing environment. This study provides evidence that microorganisms in produced fluid are revivable in laboratory incubations and retained the ability to metabolize added carbon and nitrogen substrates.

## Introduction

Hydraulic fracturing (hydrofracturing, HF), injection of pressurized fluid into the subsurface to create new fractures and to extend natural fractures, is used to extract natural gas and oil from hydrocarbon-rich geological strata. These fracture networks greatly increase the amount of hydrocarbons that can be extracted (Arthur et al., 2008; Jenkins and Li, 2008; Kargbo et al., 2010). The injected fluids are composed primarily of water and a propping agent such as sand that holds the fractures open (Nicot and Scanlon, 2012). Chemical additives increase the efficacy of the process and include viscosity modifiers, chemical stabilizers, corrosion inhibitors, and biocides (Gregory et al., 2011; Nicot and Scanlon, 2012). After a well is completed, depressurization causes 10–70% of the hydrofracturing fluid to rapidly return to the surface while the remaining fluid stays in place or flows to the surface later in production (Olmstead et al., 2013). This expelled fluid from a hydrofractured well is called “produced fluids.”

Produced fluids from hydrofractured wells are a concern because large volumes of the fluid are produced at the surface and in addition to the additives, they often contain high concentrations of salt, metals, organic compounds, radionuclides, and microorganisms due to the fluid-rock interactions at depth (Nicot and Scanlon, 2012; Barbot et al., 2013). Changes in chemical composition of produced fluids from the fluid originally injected for hydrofracturing occur due to shale dissolution, brine migration, and water-rock reactions (Blauch et al., 2009; Dresel and Rose, 2010; Warner et al., 2012). Biogeochemical activity may also change the chemical composition of produced fluids (Strong et al., 2013; Akob et al., 2015). Produced fluids must be treated for disposal or for recycling as they are important source of water for subsequent hydrofracturing fluid (Boschee, 2015); however, methods are often limited by resource availability, cost, and energy requirements (Gregory et al., 2011; Lutz et al., 2013; Rahm et al., 2013). Mitigating the compounds present in the produced fluids requires an accounting of their potential sources as well as an understanding of how they might be transformed in a hydrofractured well.

Microorganisms in the produced fluids can originate from the fractured geological formation or from anything at the surface that contacts the fluids used (e.g., water or sand used to produce hydrofracturing fluid, drilling equipment, drilling muds, well casing, gas separator) (Struchtemeyer et al., 2011; Mohan et al., 2013a,b). During hydrofracturing, the addition of chemicals such as biocides and the exposure to extreme conditions of the subsurface (high temperature, change in pH, high concentrations of salt and metals) leads to a microbial community that exhibits low diversity (Mohan et al., 2013a; Booker et al., 2017). Minor components of the community become dominant members of the produced fluids community (Mohan et al., 2013a; Cluff et al., 2014). Cluff et al. (2014) identified “indicator” genera in late stage produced fluids as being dominated by the bacterial *Halanaerobium*, unclassified *Halanaerobiaceae, Selenihalanaerobacter, Flexistipes*, and the archaeal *Methanohalophilus* and *Methanosarcinaceae*. These taxa are anaerobic halophiles that would appear to be adapted to hydrofracturing well conditions and may alter subsurface geochemistry and the legacy of the well (Cluff et al., 2014).

In order to know what long-term effects microorganisms could have on the produced fluid chemistry or in a hydrofractured well, it is important to know the fraction of these organisms that are alive in the fluids. Sulfate reducing, acid producing, and fermenting microorganisms have all been cultured from produced fluids which demonstrates their viability (Davis et al., 2012; Struchtemeyer et al., 2012; Liang et al., 2016; Booker et al., 2017); however, the ability to grow in culture may not indicate the physiological status of these cells (Rappé and Giovannoni, 2003; Puspita et al., 2012). In one metagenomic study, genes related to carbohydrate metabolism and stress response increased during the first 9 days of hydrofracturing suggesting that the microbial community responds to changes associated with hydrofracturing (Mohan et al., 2014). These investigations have clarified some aspects of the microbial ecology of the produced fluids but additional studies are needed to assess the degree to which microbes are alive in the produced fluids.

The aim of this research was to determine whether microbial communities in the produced fluids from hydrofractured systems retain the ability to assimilate carbon and nitrogen, the conditions under which such assimilation occurs, and the types of microbial communities associated with assimilation. We incubated a produced fluid for 60 days with ^13^C-labeled glucose, acetate, methanol, bicarbonate, or methane, and ^15^N-labeled ammonium chloride, all compounds that could be derived either from the subsurface or hydraulic fracturing fluid (Orberger et al., 2005; Horsfield et al., 2006; Environmental Protection Agency [EPA], 2012; Stringfellow et al., 2014). A nano-scale secondary ion mass spectrometry (NanoSIMS), a ultra-high spatial resolution isotope imaging instrument, was used to detect incorporation of stable isotopes in individual cells (Morono et al., 2011). Isotope ratio monitoring-gas chromatography-mass spectrometry, and high temperature catalytic oxidation were used to measure ^13^C-labeled metabolic products. Community analysis was conducted to determine the microbial communities that developed due to the availability of different substrates.

## Materials and Methods

### Sample Collection

Produced fluid samples were collected from the gas/liquid separator of a hydraulically fractured, horizontal well in Greene Co., Pennsylvania in May 2014. The well was drilled into the Marcellus shale which has a temperature that ranges from 35 to 51°C and an approximate pressure of 40 MPa at this location (USGS; Kargbo et al., 2010). The sample was collected in a sterile 4 L polypropylene bottle filled to capacity without headspace and shipped overnight to Oregon State University on ice.

### Experiment Setup

Forty milliliter aliquots of the produced fluid were incubated in 50 mL serum bottles that had been acid washed and combusted at 400°C for 4 h. Killed controls were autoclaved prior to allocation for 2 h at 121°C. Anoxic samples were sparged with nitrogen gas in an anaerobic chamber and capped with butyl rubber septa (Chemglass, CLS-4209-14). The absence of oxygen was confirmed by measuring oxygen saturation with a Micro TX3 – AOT Microsensor oxygen meter (PreSens, Regensburg, Germany). The supplemental carbon substrates were ^13^C-labeled-glucose (^13^C_6_, 99%), -acetate (1,2-^13^C_2_, 99%), bicarbonate (^13^C, 99%), methanol (^13^C, 99%), and methane (^13^C, 99%) and the nitrogen substrate was ^15^N-labeled ammonium chloride (^15^N, 99%) (Cambridge Isotope Laboratories), and added to final concentrations of 1 and 0.1 mM, respectively, excluding methane. For methane samples, 5 mL of gas was added with a syringe and tightly sealed with an aluminum cap afterward. No other supplemental nutrient was added. In total, 25 bottles (five substrates, four time points, one killed control) were prepared and shipped via express courier (48 h transit time) to the Kochi Institute for Core Sample Research, JAMSTEC in Kochi, Japan for downstream isotopic and genomic analysis. Once at Kochi, all samples were incubated at 25°C. Individual incubations with each supplemental carbon source were destructively sampled at 0, 19, 32, or 60 days for analyses.

### Cell Counts

To prepare samples for cell counts, serum bottles were sonicated for 30 s and briefly vortexed to remove precipitates and cells from the sides of the bottles. Twenty milliliters were transferred to a Falcon tube and centrifuged. The sediment pellets were fixed with 4% paraformaldehyde in 1 × phosphate buffered saline (PBS) for 3 h at 4°C. The pellets were washed twice with 1 × PBS and then stored in a 1:1 solution of 1 × PBS: 100% ethanol at -20°C. Samples were prepared as described in Morono et al. (2013). Briefly, fixed sediment slurry was resuspended in 2.5% NaCl and treated with a detergent mix composed of 12.5 mM EDTA, 12.5 mM Na_3_PO_4_, 0.1% v/v Tween-80, 0.1% MeOH, final concentration. Cells were detached from the produced fluid precipitates by shaking at 500 rpm for 1 h (Shake Master, Bio Medical Science) and sonicating at 200 watts in 30 s intervals for 10 cycles (Bioruptor UCD-250, Cosmo Bio). Carbonate minerals were removed with hydrofluoric acid (1.1% v/v, final concentration) for 20 min and then samples were neutralized with 1.5 M Tris-base. The final solution was added to 2.5% NaCl and filtered through 0.22 μm black polycarbonate membranes (Isopore Membrane Filter GTBP, Merck). Cells were stained with a 1:40 dilution of SYBR-green I (Thermo): 1 × TE buffer. Five hundred microliters of 1:100 diluted microspheres were added as a reference for microscopic focusing and the resulting membrane was mounted with 3–10 μL of mounting solution (1:2 mixture of TE buffer and Vectashield [Vector Laboratories]). The cells were counted with an automated fluorescence microscope (Morono and Inagaki, 2010).

### DNA Extraction and 16S rDNA Sequencing

To collect microbial community DNA, 10 mL aliquots of the produced fluids were collected after sonication and vortexing. The aliquots were centrifuged and the remaining pelleted solid was stored at -80°C. Genomic DNA was extracted using the hot alkaline method (Morono et al., 2014). In brief, pellets were warmed to 70°C in 12.5 mM EDTA for 10 min and cells were lysed at 70°C with lysis solution (1% SDS, and 1 N NaOH, final concentration) for 20 min. Supernatant was transferred to a tube with neutralization buffer (1 M N HCl, 0.3 M Tris-HCl). The remaining pellet was washed with prewarmed distilled water, centrifuged, and supernatant was transferred to the same sample tube. The extract was treated with equal volumes of phenol–chloroform–isoamyl alcohol (25:24:1) and chloroform–isoamyl alcohol (24:1) and then precipitated by adding a 1/10 volume of 3 M sodium acetate, and a 1:500 volume of polyacrylamide. The V4 region of the 16S rRNA gene was sequenced using Illumina MiSeq. The Illumina sequences were aligned to the SILVA database (release 123, Quast et al., 2013) using the program Mothur (version 1.37.0, Schloss et al., 2009). Chao richness, abundance-based coverage estimator (ACE), Jackknife, Shannon, and Simpson diversity estimators were calculated using Mothur.

The genera detected mainly constituted less than 1% of the community and were considered to be minor community. Genera that were abundant in PCR blanks or DNA negative controls and not abundant in the experimental samples (Supplementary Table 1) were deemed to be contaminants and were manually removed from the dataset. Some genera were also removed because they are often detected as contaminants during sequencing studies or because they are known to be associated with human contamination (Supplementary Table 1; Salter et al., 2014). Using this approach, approximately 5% of the sequences from all of the experimental samples were removed. However, the percent of sequences removed also varied from sample to sample (Supplementary Table 2). Two samples had notably high percentages of putative contaminants and fewer sequences as well.

### Activity Detection Using NanoSIMS

Cells were separated from sediments using a multi-layer density gradient separation protocol (Morono et al., 2013). Samples were layered over a Nycodenz and sodium polytungstate multi-layer density solution. After centrifugation, the supernatant was removed and filtered through an Anodisc membrane (GE Healthcare) followed by staining with 1:40 dilution of SYBR Green I in 1 × TE buffer. Cells were resuspended by sonicating the membrane and the resulting cell suspension was subjected to fluorescence-activated cell sorting by using Moflo Cell Sorter (Beckman Coulter). Sorted cells were captured onto an indium-tin-oxide (ITO) coated black polycarbonate membrane.

Isotopic imaging analysis was conducted with a NanoSIMS 50L ion microprobe (AMETEK Co., Ltd., CAMECA BU) at the Kochi Institute for Core Sample Research, JAMSTEC. Samples on the ITO coated polycarbonate membrane were pre-sputtered at high beam currents (30–40 pA) for a few minutes to remove surface contamination and to obtain steady state of the secondary ion intensities before measurement. The secondary ions of ^12^C^-^, ^13^C^-^, ^16^O^-^, ^12^C^14^N^-^, and ^12^C^15^N^-^ and ^32^S^-^ were collected and measured in parallel at a mass resolution of ∼9,000 that is sufficient to separate the ^13^C^-^ from the ^12^C^1^H^-^ and ^12^C^15^N^-^ from the ^13^C^14^N^-^. Detailed isotopic images of the cells were obtained by rastering a 0.8–1.3 pA 16 keV Cs^+^ primary ion beam (∼100 nmφ) over an area of 25 × 25 μm field of view. Each image consisted of 256 × 256 pixels with a dwell time of 2 ms per pixel, and the final image was created by amalgamating 20 images for same analysis area. Recorded images and data were processed using a CAMECA WinImage software and OpenMIMS plugin (Gormanns et al., 2012) in ImageJ (Schneider et al., 2012) distribution of Fiji (Schindelin et al., 2012). The different scans of each image were aligned to correct image drift during acquisition. Final images were generated by adding the secondary ion counts of each recorded secondary ion for each pixel over all scans. Intracellular carbon and nitrogen uptake from stable isotope-labeled substrates was calculated by drawing regions-of-interest on ^12^C^14^N^-^ and/or ^32^S^-^ images and calculating ^13^C/^12^C and ^15^N/^14^N ratio (inferred from the ^12^C^15^N/^12^C^14^N ratio) while having the data from blank filter area for standardizing multiple analysis data.

### Metabolic Byproducts

The metabolic byproducts of the supplemented ^13^C-labeled carbon sources, ^13^CO_2_, dissolved inorganic ^13^C, and ^13^CH_4_, were all measured with IRM–GC–MS using a Thermo/Finnigan Delta Plus XP IRMS instrument (Thermo Electron Corp., San Jose, CA, United States). Total CO_2_, dissolved inorganic carbon (DIC), and CH_4_ were also measured.

### Nucleotide Sequence Accession Numbers

The nucleotide sequences reported in this study have been deposited in the DDBJ/EMBL/GenBank database under accession number DRA007788.

## Results

### Sample Collection and Cell Counts

**FIGURE 1:**
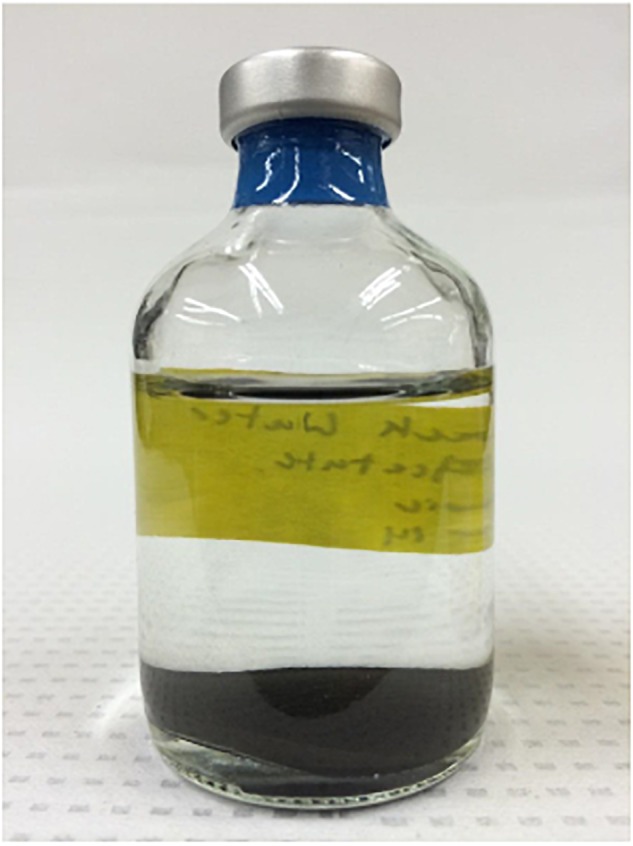
Serum bottle containing produced water as prepared for anoxic incubations.

Upon arrival at Oregon State University, the produced fluid sample was black in color. The solids at the bottom of each serum bottle (Figure 1) served as a relative indicator for the presence of oxygen wherein the anoxic incubations remained black. A sample exposed to oxygen turned orange suggesting oxidation of iron.

Cells were enumerated with automated fluorescence microscopy at 0, 19, 32, and 60 days of incubation. At the beginning of the incubation period (0 day), the cell numbers in the samples varied between 7.9 (±1.5) × 10^7^ cells/mL of the produced fluid. During the incubation period from 0 to 19 days, cell numbers declined to between 1.6 × 10^7^ and 3.5 × 10^7^ cells/mL and then, between 19 and 60 days, a gradual decline in cell numbers was observed yielding between 1.9 × 10^6^ and 3.6 × 10^6^ cells/mL by the time the experiment ended.

### Metabolic Byproducts

We measured DIC in the incubation samples at 60 days in order to determine the degree to which different substrates were respired under anoxic conditions. Samples containing ^13^C-labeled acetate, methanol, methane, and no substrate addition control contained DIC concentrations of 2.8, 2.7, 2.9, and 2.7 mM, respectively, and carbon isotopic composition (atomic % of ^13^C-DIC) of 1.19, 1.19, 1.12, and 1.10%, respectively. The killed controls all had a lower concentration of DIC with lower ^13^C (^13^C-labeled acetate: 0.9 mM, 1.12%; ^13^C-labeled methanol: 0.9 mM, 1.11%; ^13^C-labeled methane: 1.0 mM, 1.13%, we did not prepare a killed control for no substrate addition control). Although DIC in the killed control for ^13^C-labeled glucose was not measured, the sample containing ^13^C-labeled glucose had the highest concentrations of DIC and atomic % of ^13^C of 4.8 mM and 1.40%, respectively.

Total CH_4_ and carbon isotopic composition of CH_4_ (atomic % of ^13^C) in the incubation samples at 32 days and 60 days were measured to determine if any of the substrates were used for methanogenesis. The total CH_4_ at 32 days was between 0.8 and 1.6 μM for all samples. At 60 days the total methane concentrations were more varied with samples containing acetate, methanol, bicarbonate, and glucose showing evidence of 7.5, 0.9, 3.4, and 2.1 μM CH_4_, respectively. The killed controls had no detectable methane. Atomic % of ^13^C-CH_4_ values were highest in the incubation containing methanol at both 32 and 60 days with 34.54 and 36.13%, respectively. Atomic % of ^13^C-CH_4_ in the incubation with ^13^C-bicarbonate at 32 and 60 days were 3.36 and 2.08%, respectively. Atomic % of ^13^C-CH_4_ in the incubations with ^13^C-acetate or ^13^C-glucose didn’t show high values (0.997–1.002%).

### NanoSIMS Analysis

**FIGURE 2:**
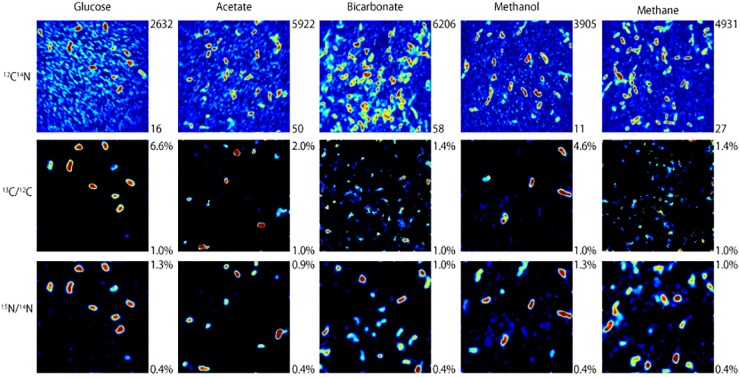
NanoSIMS images of microbial cells incubated with ^13^C-labeled carbon sources and ^15^NH_4_Cl for 32 days under anoxic conditions. The top row illustrates ^12^C^14^N ion counts that identify microbial cells. The middle and bottom rows show which of those microbial cells assimilated ^13^C and ^15^N, respectively, with warmer colors indicating a higher degree of assimilation.

NanoSIMS analysis revealed that microorganisms in the produced fluid actively assimilated carbon and nitrogen provided in the incubations. For carbon, this was indicated by an atomic % ^13^C value of ≥1 in individual cells (see Table 1 for summary of ^13^C assimilation in different treatments) and NanoSIMS images that were taken for different carbon sources under anoxic conditions. ^13^C assimilation was observed in all of the substrates other than methane (Figure 2 and Supplementary Figure 1). The relative abundance of ^13^C in individual cells is illustrated in Figure 2 (middle row) by color gradients with warmer colors indicating a higher percentage of ^13^C incorporated during the incubation and cooler colors indicating a lower abundance of ^13^C incorporated during the incubation. Glucose and methanol were assimilated favorably in the incubations. The incubation containing bicarbonate and acetate showed very slight ^13^C assimilation after 60 days. Anoxic methane samples showed no assimilation of ^13^C (Table 1).

**Table 1:** Summary of ^13^C assimilation in individual cells.

Carbon source	Time (days)	Maximum atomic^15^N% assimilation	Maximum atomic^13^C% assimilation	Number of cells that assimilated ^15^N	Number of cells that assimilated ^13^C	Total number of cells observed	Percent of cells showed substrate incorporation
Acetate	0	–	–	–	–	–	–
	19	–	–	–	–	–	–
	32	0.8	3.9	0	5	33	15
	60	0.8	4.2	0	8	30	27
Bicarbonate	0	–	–	–	–	–	–
	19	0.1	0.8	0	0	85	0
	32	0.8	0.9	0	0	100	0
	60	1.0	1.1	1	2	53	4
Glucose	0	–	–	–	–	–	–
	19	0.9	8.1	0	60	76	79
	32	1.0	6.1	4	11	14	79
	60	1.0	11.4	6	22	30	73
Methane	0	–	–	–	–	–	–
	19	–	–	–	–	–	–
	32	0.9	0.2	0	0	70	0
	60	0.9	0.3	0	0	61	0
Methanol	0	–	–	–	–	–	–
	19	0.2	0.1	0	0	54	0
	32	0.9	4.6	0	6	39	15
	60	0.5	0.4	0	0	29	0


*The minimum and maximum atomic ^*13*^C% assimilation show the range of assimilation that was observed individual cells. A dash indicates that data was not taken for that sample*.

**FIGURE 3:**
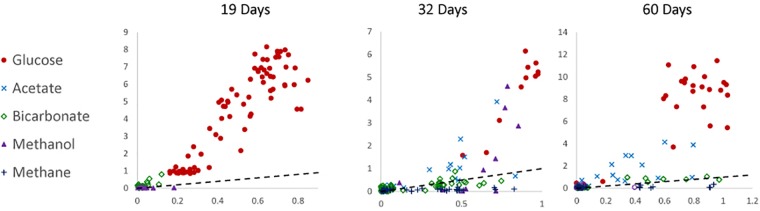
Carbon (atomic % ^13^C; *y*-axis) to nitrogen (atomic % ^15^N; *x*-axis) assimilation ratios of 19-, 32-, and 60-day incubations under anoxic conditions. The dotted line represents a 1:1 ratio. Data were not available for 0-day incubations.

To determine the relative preference that substrate-assimilating cells had for carbon or nitrogen, the assimilation ratios of ^13^C and ^15^N were plotted (Figure 3). In general, cells that were able to assimilate nutrients assimilated more ^13^C than ^15^N. Carbon from glucose was preferentially assimilated over nitrogen between 19 and 60 days. Carbon from acetate was preferentially assimilated over nitrogen at 32 and 60 days. Methanol assimilation was only observed at 32 days, but was again preferred over nitrogen. Few of the observed cells assimilated a significant amount of bicarbonate by 60 days.

### Microbial Community Characterization

**FIGURE 4:**
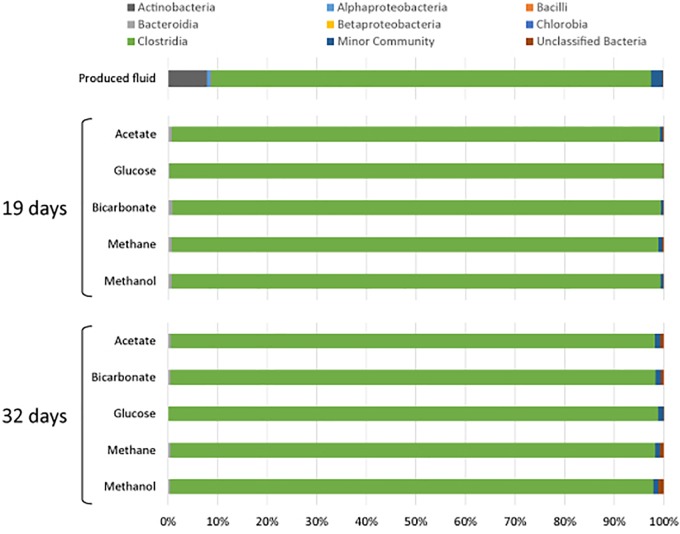
Microbial community composition of produced water after 19 and 32 days of incubation with different carbon substrates added.

**FIGURE 5:**
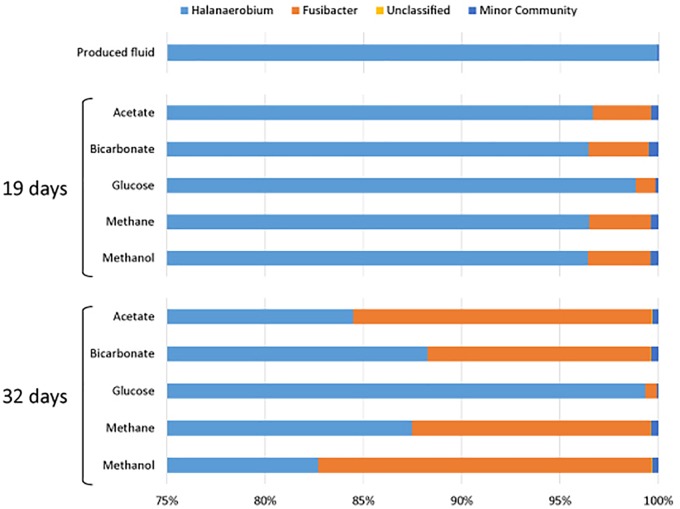
Genera of the class Clostridia that were present in produced water after 19 and 32 days of incubations with different carbon substrates added.

All samples at 19 and 32 days were dominated by the *Clostridia* such that cells from this class of Bacteria composed over 95% of the community (Figure 4). Within the *Clostridia, Halanaerobium* and *Fusibacter* were the dominant genera (Figure 5). While *Halanaerobium* composed 80% or more of the community in most incubations, *Fusibacter* became a larger portion of the community by 32 days, with the exception of samples containing glucose (Figure 5). The diversity of the microbial communities varied over time and between carbon substrates (Table 2). For example, Chao richness as an indicator of community diversity decreased between 19 and 32 days in incubations that contained acetate or methane, but increased during this same interval in incubations that contained glucose or methanol. Rarefaction curves indicated that the sequencing effort captured part of the diversity in the incubated samples of produced fluid (Supplementary Figure 2).

**Table 2:** Sequencing and diversity estimates of the produced fluids for 19 or 32 days under anoxic conditions.

Carbon source	Time (days)	Number of sequences	Observed OTUs_0.03_	Coverage (%)	Chao richness	ACE	Jackknife	Shannon	Simpson
Acetate	19	96,774	128	95	254	365	337	0.34	0.89
	32	63,893	67	97	100	177	99	0.63	0.69
Bicarbonate	19	98,732	103	97	209	343	221	0.34	0.89
	32	81,528	116	96	209	284	216	0.58	0.74
Glucose	19	89,707	57	97	99	109	104	0.12	0.96
	32	106,533	81	96	357	323	2106	0.13	0.96
Methane	19	110,675	86	96	156	209	163	0.38	0.87
	32	98,699	95	96	136	190	138	0.58	0.73
Methanol	19	84,115	88	97	145	213	154	0.33	0.89
	32	93,031	135	96	222	326	246	0.67	0.66


*All diversity estimates were made at a 97% similarity level*.

## Discussion

Our research on the metabolic activity of cells present in produced fluids is consistent with reports from past investigations that establish the role of microbes in corrosion and gas souring during hydrocarbon recovery (Enning and Garrelfs, 2014), and those specific to hydrofracturing that have characterized the microbial ecology of the produced fluids (Struchtemeyer et al., 2011; Struchtemeyer and Elshahed, 2012; Mohan et al., 2013b, 2014; Cluff et al., 2014; Akob et al., 2015; Daly et al., 2016). In our research, isotopically labeled nutrients were used to determine the survival and revivability of microorganisms derived from a fluid produced from hydrofractured shales. The carbon compounds used were those that could be available in hydrofractured wells including carbohydrates, short-chain fatty acids, C1 compounds, and inorganic carbon. Glucose, acetate, and methanol are all compounds that may be derived from chemical additives. Methanol is added as a gel stabilizer, acid corrosion inhibitor or winterizing agent (Stringfellow et al., 2014; Daly et al., 2016; FracFocus Chemical Disclosure Registry, 2016). Some hydrofracturing additives can break down into simpler carbon compounds such as glucose or acetate (Stringfellow et al., 2014). Acetate can be an important carbon source in the subsurface if formed by thermal cracking or biodegradation of complex organic compounds found in the subsurface (Horsfield et al., 2006). Methane and bicarbonate are naturally present in shale and dissolve into the produced fluids. The nitrogen source, ammonium, can be present naturally in shale as a substitute for potassium ions in illite, and present as ammonium chloride which may be added to hydrofracturing fluid as a scale inhibitor (Orberger et al., 2005; Environmental Protection Agency [EPA], 2012). We found that the produced fluid microorganisms, dominated by two genera of halophiles, were alive, revivable, and able to metabolize added compounds anabolically and catabolically under anoxic conditions.

### Metabolism of Carbon Sources Under Anoxic Conditions

To determine which carbon compounds were metabolized under anoxic conditions, the concentration and ^13^C-atomic % of DIC and CH_4_ were analyzed. DIC concentrations were higher in the no substrate added incubation (2.7 mM) than all killed control conditions (0.9∼1.0 mM) showing that the organic carbon originally present in produced fluid was metabolized by the microbial community. As discussed above, carbohydrates, short-chain fatty acids, C1 compounds, and inorganic carbon as well as nitrogen compounds such as ammonium salts and amines are all compounds that may be present in produced fluid (Stringfellow et al., 2014; FracFocus Chemical Disclosure Registry, 2016). Also, degradation of guar gum, a viscosifier used in injected fluids, by microorganism of *Halanaerobium* sp. can produce acetate (Liang et al., 2016).

The atomic % of ^13^C-DIC in the sample containing ^13^C-labeled glucose (1.40%) demonstrates the ability of microbes in the produced fluid to metabolize glucose. Fermentative glucose consumption was observed in *Halanaerobium* isolate (Booker et al., 2017). It is possible that anaerobic respiration in hydrofractured gas wells is similar to that observed in oil wells where ferrous iron or sulfate are often plentiful and commonly used as electron acceptors (Nazina et al., 1995; Röling et al., 2003; Struchtemeyer and Elshahed, 2012). Sulfate reducers are particularly troublesome in hydrofracturing wells because they produce hydrogen sulfide, which corrodes well casings and equipment (Bottero et al., 2010; Cord-Ruwisch et al., 2013; Booker et al., 2017; Lipus et al., 2017). On the other hand, fermentation has been used to enhance oil recovery through the release of metabolic byproducts such as acids, solvents, or gases which can increase access to oil and increase its mobility in the formation (Desai and Banat, 1997). We observed a 2.1 mM increase in DIC concentration (to 4.8 mM) over the no substrate addition control (2.7 mM); however, the increase of atomic % of ^13^C-DIC to 1.40% could not solely be explained by the metabolism of glucose. This suggests that, when added, glucose is metabolized as a carbon source but also may stimulate the activity of microorganisms to metabolize other organic compounds present in the produced fluid during incubation.

Incubations containing ^13^C-labeled acetate showed a significant increase of DIC concentrations and atomic % of ^13^C-DIC, which indicate anaerobic metabolism of both added acetate and other organic compounds in the produced fluid. High atomic % of ^13^C-CH_4_ was not observed in the incubation. Because acetoclastic methanogens are sensitive to high salinity environments (Waldron et al., 2007), they may be less active in hydrofractured systems than cells that possess other metabolic strategies.

There was a slight increase in atomic % of ^13^C-DIC in incubations containing ^13^C-labeled methanol. This is consistent with methanol incorporation into microbial cells, as carried out via methylotrophic metabolisms. Methylotrophs can use methane, methanol and other methylated compounds as their sole carbon and energy source (Chistoserdova, 2011). Incubations containing methanol yielded high levels of atomic % of ^13^C-CH_4_ by the end of the 60-day incubation period compared to incubations with all other substrates, indicating high methanogenesis activity using methyl moiety.

The high atomic % of ^13^C-CH_4_ values in incubations containing ^13^C-labeled bicarbonate suggest the occurrence of hydrogenotrophic methanogenesis.

### Assimilation of Carbon and Nitrogen Under Anoxic Conditions

NanoSIMS was used to view carbon and nitrogen assimilation in individual cells and to compare the utilization of different carbon sources by the microorganisms in the produced fluids under anoxic incubations. Anaerobic activity is expected as the produced fluid is derived from a highly reduced, anoxic subsurface environment. The produced fluid used in this sample had low oxygen saturation when the experiments were started. Aerobic microbial communities have been identified in hydrofracturing fluid prior to injection into the subsurface; however, facultative and strict anaerobes dominate in the fluids when they return from the subsurface (Mohan et al., 2011, 2013a; Cluff et al., 2014). Glucose was the most readily assimilated carbon source under anoxic conditions. Between 73 and 79% of the microbial cells in the incubated samples showed assimilation of glucose and the highest assimilation of ^13^C (∼11.4%) was observed. A wide variety of anaerobic microorganisms, particularly fermenters possess phosphoenolpyruvate:carbohydrate transferase systems that can transport glucose into the cell and phosphorylate it, making it directly available for catabolic or anabolic reactions (Roseman, 1969; Romano et al., 1970, 1979). This finding demonstrates the ability of microorganisms in the produced fluids to utilize carbohydrates as their carbon source and is consistent with published evidence of microbes that possess genes related to carbohydrate metabolism or microbes capable of glucose use in produced fluids (Mohan et al., 2014; Daly et al., 2016; Booker et al., 2017). Polymer gels are added as proppant carriers and viscosity modifiers; however, high temperatures, extreme chemical conditions, or biological activity can degrade them to simple carbohydrates (Kahrilas et al., 2015). For example, guar gum is added in high concentrations to hydrofracturing fluid to adjust viscosity, and its degraded byproducts promote microbial activity downhole (Lester et al., 2014).

Acetate was the next most readily assimilated carbon source (Table 1 and Figure 3) and is a common byproduct of fermentation in the subsurface (Lovley and Chapelle, 1995). Acetic acid, which is added to hydrofracturing fluid as a buffer, could also be a source of carbon (Stringfellow et al., 2014). Fermentative production of acetate was observed in a microcosm experiment dominated by *Halanaerobium* (Borton et al., 2018). Acetate can be assimilated by the glyoxylate cycle, alternate glyoxylate pathway, and glyoxylate regeneration cycle (Ensign, 2006) and is used by iron, sulfate, and nitrate reducing bacteria, all of which have been identified in the produced fluids from gas wells (Struchtemeyer and Elshahed, 2012; Mohan et al., 2013a,b). Acetoclastic methanogens can also assimilate acetate, but their presence may be unlikely in this setting because of their sensitivity to high salinity and to changes in salt concentration (Patel and Roth, 1977; Waldron et al., 2007; Winkel et al., 2014).

A particularly interesting result of our study was that microorganisms in the produced fluid assimilated carbon from methanol, a compound sometimes added to hydrofracturing fluid as a winterizer or chemical stabilizer. This result was observed after 32 days of incubation and not again at 60 days; however, such cells may have been present but below the limit of detection using NanoSIMS. This may suggest the presence of methylotrophs, which could also include methanotrophs and methanogenic archaea. We did not observe either anabolic or catabolic metabolism of methane during our study. This may have resulted because ^13^CH_4_ was added as a gas without pressurization and was therefore less accessible by microorganisms. Methanotrophy should not be ruled out; however, rates of anaerobic oxidation of methane can be quite low (c.f., Orcutt et al., 2013) and may not have been detected during our relatively short incubations. Methane in the subsurface is dissolved into hydraulic fracturing fluid under pressure, increasing its solubility and making it readily available.

Carbon fixation was not significantly observed in cells incubated with bicarbonate. The maximum percentage of cells that were observed to assimilate ^13^C-bicarbonate was only 1% at 60 days, but cells that assimilated bicarbonate also assimilated small amounts of nitrogen. This may suggest that autotrophy is not an energy efficient metabolism for microorganisms in hydrofracturing wells, at least under the conditions of our experiment. Ample carbonate (as calcium carbonate) often exists as natural fractures in shale, and can dissolve into solution upon the addition of hydrofracturing fluid (Gale and Holder, 2010). Sodium or potassium carbonate may be added to hydrofracturing fluid (Stringfellow et al., 2014) such that concentrations reach tens to hundreds of mg/L (Haluszczak et al., 2013; Lester et al., 2015). Produced fluids from the Marcellus shale contains an average of 165 mg/L of calcium carbonate (Barbot et al., 2013).

The ratios of ^13^C and ^15^N assimilation into microorganisms in the produced fluid showed that cells assimilating supplied nutrients incorporated more carbon than nitrogen although DIC measurements clearly showed metabolism of other non-labeled sources of carbon or nitrogen present in the produced fluid. This apparent preference for carbon was particularly noticeable in glucose incubations. This may be because nitrogen is more limiting than carbon in such environments, or alternatively that these microorganisms were incorporating high C/N ratio organic matter rather than ammonium. In our study, it seems unlikely that the produced fluid is carbon limited, considering the high organic content of shale. The C/N ratio of Devonian (Marcellus shale) age organic carbon is between 174.80 and 193.28 atomic C/N, and the C/N ratio of kerogen is even higher, making nitrogen the limiting nutrient (Orberger et al., 2005). Produced fluids from Marcellus shale were reported to have total organic carbon concentrations of between 1.2 and 1530 mg/L (Barbot et al., 2013). Also considering the length of these incubations, the incorporation of ^13^C may have not come from the original carbon source but rather from cell debris that had previously incorporated the ^13^C, which may have served as an additional nitrogen source. In this interpretation, there may have been some turnover of the originally incorporated labeled substrates. Heterotrophic bacteria can assimilate amino acids over ammonium as this allows them to conserve energy required to build amino acids (Kirchman et al., 1989; Morono et al., 2011).

Interpretation of microbial activity, including carbon and nitrogen assimilation, can be confounded by environmental conditions such as high salinity, high temperature, high pressure, or non-neutral pH that are common in subsurface environments (Postma and Jakobsen, 1996; Colwell et al., 1997; Fredrickson et al., 1997). Hydrofracturing produces a dynamic environment where these conditions vary depending on the stage of hydrofracturing and the residence time of the fluid downhole. Microbial cells may not be assimilating carbon and nitrogen for growth and replication, but may instead assimilate these elements mainly for cell maintenance. Extreme conditions increase the amount of energy that microorganisms need to repair or replace cellular components even if they are well adapted to the environment (Hoehler, 2007; Morozkina et al., 2010). Furthermore, slow growth could be advantageous for cells in hydrofractured wells where biocides are used because slow growth and nutrient limitation can reduce microbial sensitivity to antimicrobials, which are commonly used in hydrofracturing (Mah and O’Toole, 2001).

### Microbial Communities in Produced Fluid Incubations

Microbial community diversity determinations were conducted on the produced fluid samples incubated under anoxic conditions for 0, 19, and 32 days. The 0 day glucose sample had 50% of the sequences removed and this was also apparent in the microbial community, but less so than the 0 day no substrate control. It is suspected that these samples had poorer DNA recovery and PCR amplification than other samples.

Microbial diversity estimates indicated that the diversity in these samples was lower than what has been previously found in produced fluids and impoundment waters (Struchtemeyer et al., 2012; Mohan et al., 2013a,b). This may have resulted from conditions when the samples were collected, sample handling, or from the placement of these microbial communities into microcosms thereby eliminating the input and output of nutrients and metabolic waste products. Most of the microbial communities in this experiment were very similar, indicating that the addition of substrate did not affect the microbial community. The composition of the microbial community was similar to what has been observed in late stage produced fluids (Mohan et al., 2013a; Cluff et al., 2014; Liang et al., 2016; Booker et al., 2017).

All of the samples were dominated by Clostridia. This is in agreement with past observations wherein late stage produced fluids were dominated by *Clostridia*, specifically *Halanaerobium* (Mohan et al., 2013a; Cluff et al., 2014). Cluff et al. (2014) also reported other indicator genera that we detected including *Flexistipes*, and *Methanohalophilus*. *Halanaerobium* accounted for 80% or more of the *Clostridia* sequences in all of the incubations. *Halanaerobium* are anaerobic, halophilic, alkalophilic, and thermophilic bacteria and thus likely to be adapted to oil and gas wells environments. For example, *H. locusroseus* can grow in 5–30% NaCl, and at 20–50°C (Cayol et al., 1995). *H. congolense* in particular has been isolated from a number of oil wells and implicated in well corrosion due to hydrogen sulfide production by thiosulfate reduction (Magot et al., 2000; Ravot et al., 2006). *Halanaerobium* can use carbohydrates as carbon sources and therefore may have been responsible for the assimilation of glucose under anoxic conditions (Oren, 2002). As a fermenter, *Halanaerobium* produces acetate and CO_2_, compounds that may sustain other microorganisms. *Halanaerobium* spp. have previously been detected in hydrofracturing fluids from separators, storage tanks and impoundments (Davis et al., 2012; Mohan et al., 2013a,b; Akob et al., 2015; Liang et al., 2016; Booker et al., 2017; Lipus et al., 2017).

*Fusibacter* was a prominent member of the microbial communities throughout this experiment. *Fusibacter* are also fermenters and can produce acetate by fermenting glucose (Ravot et al., 1999). This genus has been isolated from many hydrocarbon-rich environments and can produce hydrogen sulfide by reducing thiosulfate or elemental sulfur (Ravot et al., 1999; Agrawal et al., 2010; Ben Hania et al., 2012; Smii et al., 2015). Its role in carbon assimilation is likely similar to that of *Halanaerobium* in that it may use glucose as a carbon or energy source. Interestingly, *Fusibacter* began to increase in percentage of the community between 0 and 32 days, as did the number of sequences. Possibly, *Fusibacter* was less sensitive to conditions (e.g., product inhibition) in the microcosms than *Halanaerobium*. For example, the growth of *H. saccharolyticum* can be inhibited by an excess of acetate (Kivistö et al., 2012). Because *Halanaerobium* is an alkaliphile, the production of hydrogen during fermentation may have caused a reduction in pH, making the produced fluids environment in a closed system less habitable. However, some species of *Fusibacter* are also prone to product inhibition by hydrogen, unless thiosulfate is present (Ravot et al., 1999). The common finding of both *Fusibacter* and *Halanaerobium* in oil and gas reservoirs suggests that additional research should be conducted on their presence and potential interactions in these environments.

*Halanaerobium* and *Fusibacter* were the most abundant microorganisms, but their presence does not explain metabolic processes observed with carbon sources other than glucose. Minor members of the community may have played a larger part in metabolizing other carbon sources. Appearing in much lower abundance were the genera *Anaerophaga, Geotoga, Flexistipes, Asticcacaulis, Carboxydocella, Desulfotomaculum, Methanohalophilus*, and a number of unclassified microorganisms. Most of these organisms are halophilic, thermophilic, or both, and should be well adapted to gas well conditions. Representatives of *Anaerophaga* and *Geotoga* can ferment glucose and may have also contributed to respiration or assimilation of glucose (Davey et al., 1993; Denger et al., 2002). *Flexistipes*, a thermophile which requires at least 3% NaCl to grow, can utilize acetate as an energy source (Fiala et al., 1990; Nakano and Zuber, 2004). The presence of *Carboxydocella* is intriguing as it is known for its use of carbon monoxide (Sokolova et al., 2002; Slepova et al., 2006) which may be present in shales and may be removed by the produced fluid along with methane to the surface. *Desulfotomaculum* spp. are sulfate reducers often found in oil wells, and one species, *D. kuznetsovii*, is methylotrophic (Nazina et al., 1988; Nilsen et al., 1996). Recent reconstruction of the genome of *Methanohalophilus* from produced shale fluids indicated methanogenesis using methanol via methanol:MtaC co-methyltransferase (*MtaB*) (Daly et al., 2016). *Methanohalophilus* was detected in all of our incubations; however, the highest atomic % of ^13^C-CH_4_ was observed in the methanol-amended incubation (34.54 and 36.13% at 32 and 60 days), strongly indicating methanogenesis by *Methanohalophilus* in our incubations. These findings are consistent with evidence presented by others showing a prominent role for microbes of this genus in produced fluids (Daly et al., 2016; Borton et al., 2018) and for their ability to use methylated compounds (Mathrani et al., 1988).

## Conclusion

We found that microorganisms in incubations of produced fluid from a hydrofractured shale were revivable in laboratory incubations and retained the ability to metabolize added substrates catabolically and anabolically. When incubated anoxically, microbial communities were active and responsive to diverse carbon substrates. Following anoxic incubations, microbial communities were not diverse and many of the genera observed were related to microbes that have previously been detected in hydraulically fractured shale fluids or similar environments and that possess qualities such as resistance to high salinity, heat, and the presence of metals. An ideal sample set to examine microbial properties of shales would involve collecting and analyzing subsurface cores with attention to sampling technique to prevent microbial contamination. Such an effort would verify whether microbes that we studied and that others have detected exist in and are active in these formations. The microbes that we examined in the produced fluid were at least fractionally alive and may have the metabolic potential to affect subsurface geochemistry and to consume organics found in the subsurface or delivered during the hydraulic fracturing process.

## Author Contributions

JW, MT, CV, and FC designed the project. JW collected the samples. JW, YM, MI, AI, TH, and TT conducted the experiments, analyzed the samples, and interpreted the data. JW, YM, AI, FI, and FC wrote the manuscript.

## Disclaimer

This report was prepared as an account of work sponsored by an agency of the United States Government. Neither the United States Government nor any agency thereof, nor any of their employees, makes any warranty, express or implied, or assumes any legal liability or responsibility for the accuracy, completeness, or usefulness of any information, apparatus, product, or process disclosed, or represents that its use would not infringe privately owned rights. Reference therein to any specific commercial product, process, or service by trade name, trademark, manufacturer, or otherwise does not necessarily constitute or imply its endorsement, recommendation, or favoring by the United States Government or any agency thereof. The views and opinions of authors expressed therein do not necessarily state or reflect those of the United States Government or any agency thereof.

## Conflict of Interest Statement

The authors declare that the research was conducted in the absence of any commercial or financial relationships that could be construed as a potential conflict of interest.

## References

[B1] Agrawal A. Vanbroekhoven K. Lal B. (2010). Diversity of culturable sulfidogenic bacteria in two oil-water separation tanks in the north-eastern oil fields of India. *Anaerobe* 16 12–18. 10.1016/j.anaerobe.2009.04.005 19427389

[B2] Akob D. M. Cozzarelli I. M. Dunlap D. S. Rowan E. L. Lorah M. M. (2015). Organic and inorganic composition and microbiology of produced waters from pennsylvania shale gas wells. *Appl. Geochem.* 60 116–125. 10.1016/j.apgeochem.2015.04.011

[B3] Arthur J. D. Bohm B. Coughlin B. Layne M. (2008). *Hydraulic Fracturing Considerations For Natural Gas Well of the Fayetteville Shale.* Available at: http://www.aogc2.state.ar.us/OnlineData/reports/ALL FayettevilleFrac FINAL.pdf (Accessed March 10, 2016).

[B4] Barbot E. Vidic N. S. Gregory K. B. Vidic R. D. (2013). Spatial and temporal correlation of water quality parameters of produced waters from Devonian-age shale following hydraulic fracturing. *Environ. Sci. Technol.* 47 2562–2569. 10.1021/es304638h 23425120

[B5] Ben Hania W. Fraj B. Postec A. Fadhlaoui K. Hamdi M. Ollivier B. (2012). Fusibacter tunisiensis sp. nov., isolated from an anaerobic reactor used to treat olive-mill wastewater. *Int. J. Syst. Evol. Microbiol.* 62 1365–1368. 10.1099/ijs.0.034603-0 21828014

[B6] Blauch M. E. Myers R. R. Moore T. Lipinski B. A. Houston N. A. (2009). *Marcellus Shale Post-Frac Flowback Waters - Where Is All The Salt Coming From and What are the Implications? in SPE Eastern Regional Meeting.* Charleston: Society of Petroleum Engineers 10.2118/125740-MS

[B7] Booker A. E. Borton M. A. Daly R. A. Welch S. A. Nicora C. D. Hoyt D. W. (2017). Sulfide generation by dominant *Halanaerobium* microorganisms in hydraulically fractured shales. *mSphere* 2 e00257–e00317. 10.1128/mSphereDirect.00257-17 28685163 PMC5497025

[B8] Borton M. A. Hoyt D. W. Roux S. Daly R. A. Welch S. A. Nicora C. D. (2018). Coupled laboratory and field investigations resolve microbial interactions that underpin persistence in hydraulically fractured shales. *Proc. Natl. Acad. Sci. U S A.* 115 E6585–E6594. 10.1073/pnas.1800155115 29941576 PMC6048472

[B9] Boschee P. (2015). Produced and flowback water recycling and reuse: economics, limitations, and technology. *Oil Gas Facil.* 3 16–21. 10.2118/0214-0016-OGF

[B10] Bottero S. Picioreanu C. Delft T. U. Enzien M. Control D. M. Loosdrecht M. V. (2010). “Formation damage and impact on gas flow caused by biofilms growing within proppant packing used in hydraulic fracturing,” in *Proceedings of the SPE International Symposium on Formation Damage Control, SPE-128066*, (Lafayette: Society of Petroleum Engineers). 10.2118/128066-MS

[B11] Cayol J. L. Ollivier B. Patel B. K. Ageron E. Grimont P. A. Prensier G. (1995). *Haloanaerobium lacusroseus* sp. nov., an extremely halophilic fermentative bacterium from the sediments of a hypersaline lake. *Int. J. Syst. Bacteriol.* 45 790–797. 10.1099/00207713-45-4-790 7547301

[B12] Chistoserdova L. (2011). Modularity of methylotrophy, revisited. *Environ. Microbiol.* 13 2603–2622. 10.1111/j.1462-2920.2011.02464.x 21443740

[B13] Cluff M. A. Hartsock A. MacRae J. D. Carter K. Mouser P. J. (2014). Temporal changes in microbial ecology and geochemistry in produced water from hydraulically fractured marcellus shale gas wells. *Environ. Sci. Technol.* 48 6508–6517. 10.1021/es501173p 24803059

[B14] Colwell F. S. Onstott T. C. Delwiche M. E. Chandler D. Fredrickson J. K. Yao Q.-J. (1997). Microorganisms from deep, high temperature sandstones: constraints on microbial colonization. *FEMS Microbiol. Rev.* 20 425–435. 10.1111/j.1574-6976.1997.tb00327.x

[B15] Cord-Ruwisch R. Kleinitz W. Widdel F. (2013). Sulfate-reducing bacteria and their activities in oil production. *J. Pet. Technol.* 39 97–106. 10.2118/13554-PA

[B16] Daly R. A. Borton M. A. Wilkins M. J. Hoyt D. W. Kountz D. J. Wolfe R. A. (2016). Microbial metabolisms in a 2.5-km-deep ecosystem created by hydraulic fracturing in shales. *Nat. Microbiol.* 1:16146. 10.1038/nmicrobiol.2016.146 27595198

[B17] Davey M. E. Wood W. A. Key R. Nakamura K. Stahl D. A. (1993). Isolation of three species of geotoga and petrotoga: two new genera, representing a new lineage in the bacterial line of descent distantly related to the “thermotogales.”. *Syst. Appl. Microbiol.* 16 191–200. 10.1016/S0723-2020(11)80467-4

[B18] Davis J. P. Struchtemeyer C. G. Elshahed M. S. (2012). Bacterial communities associated with production facilities of two newly drilled thermogenic natural gas wells in the barnett shale (texas, USA). *Microb. Ecol.* 64 942–954. 10.1007/s00248-012-0073-3 22622766

[B19] Denger K. Warthmann R. Ludwig W. Schink B. (2002). Anaerophaga thermohalophila gen. nov., sp. nov., a moderately thermohalophilic, strictly anaerobic fermentative bacterium. *Int. J. Syst. Evol. Microbiol.* 52 173–178. 10.1099/00207713-52-1-173 11837300

[B20] Desai J. Banat I. (1997). Microbial production of surfactants and their commercial potential. *Microbiol. Mol. Biol. Rev.* 61 47–64. 9106364 10.1128/mmbr.61.1.47-64.1997 PMC232600

[B21] Dresel E. Rose A. W. (2010). *Chemistry and Origin of Oil and Gas Well Brines in Western PENNSYLVANIA. Pennsylvania Geological Survey*, 4th series Open-File Report OFOG 10-01.0. State College, PA, Penn State University.

[B22] Enning D. Garrelfs J. (2014). Corrosion of iron by sulfate-reducing bacteria: new views of an old problem. *Appl. Environ. Microbiol.* 80 1226–1236. 10.1128/AEM.02848-13 24317078 PMC3911074

[B23] Ensign S. A. (2006). Revisiting the glyoxylate cycle: alternate pathways for microbial acetate assimilation. *Mol. Microbiol.* 61 274–276. 10.1111/j.1365-2958.2006.05247.x 16856935

[B24] Environmental Protection Agency [EPA]. (2012). *Study of the Potential Impacts of Hydraulic Fracturing On Drinking Water Resources.* Progress Report. (EPA/601/R-12/011). Washington, DC: U.S.Environmental Protection Agency, Office of Research and Development.

[B25] Fiala G. Woese C. R. Langworthy T. A. Stetter K. O. (1990). Flexistipes sinusarabici, a novel genus and species of eubacteria occurring in the atlantis II deep brines of the red sea. *Arch. Microbiol.* 154 120–126. 10.1007/BF00423320

[B26] FracFocus Chemical Disclosure Registry. (2016). *FracFocus 3.0.* Available at: http://fracfocus.org/ (Accessed January 14, 2016).

[B27] Fredrickson J. K. McKinley J. P. Bjornstad B. N. Long P. E. Ringelberg D. B. White D. C. (1997). Pore-size constraints on the activity and survival of subsurface bacteria in a late cretaceous shale-sandstone sequence. northwestern new mexico. *Geomicrobiol. J.* 14 183–202. 10.1080/01490459709378043

[B28] Gale J. F. W. Holder J. (2010). Natural fractures in some US shales and their importance for gas production. *Petrol. Geol. Conf. Proc.* 7 1131–1140. 10.1144/0071131

[B29] Gormanns P. Reckow S. Poczatek J. C. Turck C. W. Lechene C. (2012). Segmentation of Multi-Isotope Imaging Mass Spectrometry Data for Semi-Automatic Detection of Regions of Interest. *PLoS One* 7:e30576. 10.1371/journal.pone.0030576 22347386 PMC3276494

[B30] Gregory K. B. Vidic R. D. Dzombak D. (2011). Water management challenges associated with the production of shale gas by hydraulic fracturing. *Elements* 7 181–186. 10.2113/gselements.7.3.181 28159795

[B31] Haluszczak L. O. Rose A. W. Kump L. R. (2013). Geochemical evaluation of flowback brine from marcellus gas wells in pennsylvania. *U S A. Appl. Geochem.* 28 55–61. 10.1016/j.apgeochem.2012.10.002

[B32] Hoehler T. M. (2007). An energy balance concept for habitability. *Astrobiology* 7 824–838. 10.1089/ast.2006.0095 18163865

[B33] Horsfield B. Schenk H. Zink K. Ondrak R. Dieckmann V. Kallmeyer J. (2006). Living microbial ecosystems within the active zone of catagenesis: implications for feeding the deep biosphere. *Earth Planet. Sci. Lett.* 246 55–69. 10.1016/j.epsl.2006.03.040

[B34] Jenkins C. D. Li C. M. B. (2008). Coalbed- and shale-gas reservoirs. *J. Petrol. Technol.* 60 92–99. 10.2118/103514-JPT

[B35] Kahrilas G. Blotevogel J. Stewart P. S. Borch T. (2015). Biocides in hydraulic fracturing fluids: a critical review of their usage, mobility, degradation, and toxicity. *Environ. Sci. Technol.* 49 16–32. 10.1021/es503724k 25427278

[B36] Kargbo D. M. Wilhelm R. G. Campbell D. J. (2010). Natural gas plays in the marcellus shale: challenges and potential opportunities. *Environ. Sci. Technol.* 44 5679–5684. 10.1021/es903811p 20518558

[B37] Kirchman D. L. Keil R. G. Wheeler P. A. (1989). The effect of amino acids on ammonium utilization and regeneration by heterotrophic bacteria in the subarctic pacific. *Deep. Res.* 36 1763–1776. 10.1016/0198-0149(89)90071-X

[B38] Kivistö A. Santala V. Karp M. (2012). 1,3-propanediol production and tolerance of a halophilic fermentative bacterium, *Halanaerobium saccharolyticum* subsp. *saccharolyticum*. *J. Biotechnol.* 158 242–247. 10.1016/j.jbiotec.2011.10.013 22085971

[B39] Lester Y. Ferrer I. Thurman E. M. Sitterley K. Korak J. Aiken G. (2015). Characterization of hydraulic fracturing flowback water in colorado: implications for water treatment. *Sci. Total Environ.* 51 637–644. 10.1016/j.scitotenv.2015.01.043 25658325

[B40] Lester Y. Yacob T. Morrissey I. Linden K. G. (2014). Can we treat hydraulic fracturing flowback with a conventional biological process? The case of guar gum. *Environ. Sci. Technol. Lett.* 1 133–136. 10.1021/ez4000115

[B41] Liang R. Davidova I. A. Marks C. R. Stamps B. W. Harriman B. H. Stevenson B. S. (2016). Metabolic capability of a predominant *Halanaerobium* sp. in hydraulically fractured gas wells and its implication in pipeline corrosion. *Front. Microbiol.* 7:988. 10.3389/fmicb.2016.00988 27446028 PMC4916785

[B42] Lipus D. Vikram A. Ross D. Bain D. Gulliver D. Hammack R. (2017). Predominance and metabolic potential of *Halanaerobium* spp. in produced water from hydraulically fractured marcellus shale wells. *Appl. Environ. Microbiol.* 83 e2659–e2716. 10.1128/AEM.02659-16 28159795 PMC5377500

[B43] Lovley D. R. Chapelle F. H. (1995). Deep subsurface microbial processes. *Rev. Geophys.* 33 365–381. 10.1029/95RG01305

[B44] Lutz B. D. Lewis A. N. Doyle M. W. (2013). Generation, transport, and disposal of wastewater associated with marcellus shale gas development. *Water Resour. Res.* 49 647–656. 10.1002/wrcr.20096

[B45] Magot M. Ollivier B. Patel B. K. C. (2000). Microbiology of petroleum reservoirs. *Antonie Van Leeuwenhoek* 77 103–116. 10.1023/A:1002434330514 10768470

[B46] Mah T.-F. C. O’Toole G. A. (2001). Mechanisms of biofilm resistance to antimicrobial agents. *Trends Microbiol.* 9 34–39. 10.1016/S0966-842X(00)01913-2 11166241

[B47] Mathrani I. M. Boone D. R. Mah R. A. Fox G. E. Lau P. P. (1988). *Methanohalophilus zhilinae* sp. nov., an alkaliphilic, halophilic, methylotrophic methanogen. *Int. J. Syst. Bacteriol.* 38 139–142. 10.1099/00207713-38-2-139 11540079

[B48] Mohan A. M. Bibby K. J. Lipus D. Hammack R. W. Gregory K. B. (2014). The functional potential of microbial communities in hydraulic fracturing source water and produced water from natural gas extraction characterized by metagenomic sequencing. *PLoS One* 9:e107682. 10.1371/journal.pone.0107682 25338024 PMC4206270

[B49] Mohan A. M. Gregory K. Vidic R. Miller P. Hammack R. (2011). “Characterization of microbial diversity in treated and untreated flowback water impoundments from gas fracturing operations,” in *Proceedings of the SPE Annual Technical Conference and Exhibition*. SPE-147414-MS, (Denver: Society of Petroleum Engineers).

[B50] Mohan A. M. Hartsock A. Bibby K. J. Hammack R. W. Vidic R. D. Gregory K. B. (2013a). Microbial community changes in hydraulic fracturing fluids and produced water from shale gas extraction. *Environ. Sci. Technol.* 47 13141–13150. 10.1021/es402928b 24088205

[B51] Mohan A. M. Hartsock A. Hammack R. W. Vidic R. D. Gregory K. B. (2013b). Microbial communities in flowback water impoundments from hydraulic fracturing for recovery of shale gas. *FEMS Microbiol. Ecol.* 86 567–580. 10.1111/1574-6941.12183 23875618

[B52] Morono Y. Inagaki F. (2010). Automatic slide-loader fluorescence microscope for discriminative enumeration of subseafloor life. *Sci. Drill.* 9 32–36. 10.5194/sd-9-32-2010

[B53] Morono Y. Terada T. Hoshino T. Inagaki F. (2014). Hot-alkaline DNA extraction method for deep-subseafloor archaeal communities. *Appl. Environ. Microbiol.* 80 1985–1994. 10.1128/AEM.04150-13 24441163 PMC3957647

[B54] Morono Y. Terada T. Kallmeyer J. Inagaki F. (2013). An improved cell separation technique for marine subsurface sediments: applications for high-throughput analysis using flow cytometry and cell sorting. *Environ. Microbiol.* 15 2841–2849. 10.1111/1462-2920.12153 23731283 PMC3910163

[B55] Morono Y. Terada T. Nishizawa M. Ito M. Hillion F. Takahata N. (2011). Carbon and nitrogen assimilation in deep subseafloor microbial cells. *Proc. Natl. Acad. Sci.* 108 18295–18300. 10.1073/pnas.1107763108 21987801 PMC3215001

[B56] Morozkina E. V. Slutskaia E. S. Fedorova T. V. Tugaǐ T. I. Golubeva L. I. Koroleva O. V. (2010). Extremophilic microorganisms: biochemical adaptation and biotechnological application (review). *Prikl. Biokhim. Mikrobiol.* 46 5–20. 10.1134/S0003683810010011 20198911

[B57] Nakano M. M. Zuber P. (2004). *Strict and Facultative Anaerobes: Medical and Environmental Aspects.* Wymondham: CRC Press 10.1201/9781482292503

[B58] Nazina T. Ivanova A. Kanchaveli L. Rozanova E. (1988). *Desulfotomaculum kuznetsovii* sp. nov., a new spore-forming thermophilic methylotrophic sulfate-reducing bacterium. *Mikrobiologiya* 57 823–827.

[B59] Nazina T. N. Ivanova A. E. Golubeva O. V. Ibatullin R. R. Belyaev S. S. Ivanov M. V. (1995). Occurrence of sulfate- and iron-reducing bacteria in stratal waters of the romashkinskoe oil field. *Microbiology* 64 245–251. 7616880

[B60] Nicot J.-P. Scanlon B. R. (2012). Water use for shale gas production in texas. *U.S. Environ. Sci. Technol.* 46 3580–3586. 10.1021/es204602t 22385152

[B61] Nilsen R. K. Torsvik T. Lien T. (1996). Desulfotomaculum *thermocisternum* sp. nov., a sulfate reducer isolated from a hot north sea oil reservoir. *Int. J. Syst. Bacteriol.* 46 397–402. 10.1099/00207713-46-2-397

[B62] Olmstead S. M. Muehlenbachs L. Shih J. Chu Z. Krupnick A. J. (2013). Shale gas development impacts on surface water quality in pennsylvania. *Proc. Natl. Acad. Sci. U SA.* 110 4962–4967. 10.1073/pnas.1213871110 23479604 PMC3612605

[B63] Orberger B. Gallien J.-P. Pinti D. L. Fialin M. Daudin L. Gröcke D. R. (2005). Nitrogen and carbon partitioning in diagenetic and hydrothermal minerals from paleozoic black shales, (selwyn basin, yukon territories, canada). *Chem. Geol.* 218 249–264. 10.1016/j.chemgeo.2005.01.012

[B64] Orcutt B. N. LaRowe D. E. Biddle J. F. Colwell F. S. Glazer B. T. Reese B. K. (2013). Microbial activity in the marine deep biosphere: progress and prospects. *Front. Microbiol.* 4:189. 10.3389/fmicb.2013.00189 23874326 PMC3708129

[B65] Oren A. (2002). *Halophilic Microorganisms and their Environments.* Dordrecht: Kluwer Scientific Publishers 10.1007/0-306-48053-0

[B66] Patel G. B. Roth L. A. (1977). Effect of sodium chloride on growth and methane production of methanogens. *Can. J. Microbiol.* 23 893–897. 10.1139/m77-131 884626

[B67] Postma D. Jakobsen R. (1996). Redox zonation: equilibrium constraints on the Fe(III)/SO4-reduction interface. *Geochim. Cosmochim. Acta* 60 3169–3175. 10.1016/0016-7037(96)00156-1

[B68] Puspita I. D. Kamagata Y. Tanaka M. Asano K. Nakatsu C. H. (2012). Are uncultivated bacteria really uncultivable? *Microb. Environ.* 27 356–366. 10.1264/jsme2.ME12092 PMC4103542 23059723

[B69] Quast C. Pruesse E. Yilmaz P. Gerken J. Schweer T. Yarza P. (2013). The SILVA ribosomal RNA gene database project: improved data processing and web-based tools. *Nucl. Acids Res.* 41 D590–D596. 10.1093/nar/gks1219 23193283 PMC3531112

[B70] Rahm B. G. Bates J. T. Bertoia L. R. Galford A. E. Yoxtheimer D. A. Riha S. J. (2013). Wastewater management and marcellus shale gas development: Trends. drivers, and planning implications. *J. Environ. Manag.* 120 105–113. 10.1016/j.jenvman.2013.02.029 23507249

[B71] Rappé M. S. Giovannoni S. J. (2003). The uncultured microbial majority. *Annu. Rev. Microbiol.* 57 369–394. 10.1146/annurev.micro.57.030502.090759 14527284

[B72] Ravot G. Magot M. Fardeau M.-L. Patel B. K. C. Thomas P. Garcia J.-L. (1999). *Fusibacter paucivorans* gen. nov., sp. nov., an anerobic, thiosulfate-reducing bacterium from an oil-producing well. *J. Syst. Bacteriol.* 49 1141–1147. 10.1099/00207713-49-3-1141 10425772

[B73] Ravot G. Magot M. Ollivier B. Patel B. K. Ageron E. Grimont P. A. (2006). *Haloanaerobium congolense* sp. nov., an anaerobic, moderately halophilic, thiosulfate- and sulfur-reducing bacterium from an African oil field. *FEMS Microbiol. Lett.* 147 81–88. 10.1111/j.1574-6968.1997.tb10224.x 9037768

[B74] Röling W. F. M. Head I. M. Larter S. R. (2003). The microbiology of hydrocarbon degradation in subsurface petroleum reservoirs: perspectives and prospects. *Res. Microbiol.* 154 321–328. 10.1016/S0923-2508(03)00086-X 12837507

[B75] Romano A. H. Eberhard S. J. Dingle S. L. McDowell T. D. (1970). Distribution of the phosphoenolpyruvate:glucose phosphotransferase system in bacteria. *J. Bacteriol.* 104 808–813. 5489437 10.1128/jb.104.2.808-813.1970 PMC285062

[B76] Romano A. H. Trifone J. D. Brustolon M. (1979). Distribution of the phosphoenolpyruvate:glucose phosphotransferase system in fermentative bacteria. *J. Bacteriol.* 139 93–97. 457606 10.1128/jb.139.1.93-97.1979 PMC216831

[B77] Roseman S. (1969). The transport of carbohydrates by a bacterial phosphotransferase system. *J. Gen. Physiol.* 54 138–184. 10.1085/jgp.54.1.138 19873641 PMC2225901

[B78] Salter S. J. Cox M. J. Turek E. M. Calus S. T. Cookson W. O. Moffatt M. F. (2014). Reagent and laboratory contamination can critically impact sequence-based microbiome analyses. *BMC Biol.* 12:87. 10.1186/s12915-014-0087-z 25387460 PMC4228153

[B79] Schindelin J. Arganda-Carreras I. Frise E. Kaynig V. Longair M. Pietzsch T. (2012). Fiji: an open-source platform for biological-image analysis. *Nat. Methods* 9 676–682. 10.1038/nmeth.2019 22743772 PMC3855844

[B80] Schneider C. A. Rasband W. S. Eliceiri K. W. (2012). NIH Image to ImageJ: 25 years of image analysis. *Nat. Methods* 9 671–675. 10.1038/nmeth.2089 22930834 PMC5554542

[B81] Schloss P. D. Westcott S. L. Ryabin T. Hall J. R. Hartmann M. Hollister E. B. (2009). Introducing mothur: open-source, platform-independent, community-supported software for describing and comparing microbial communities. *Appl. Environ. Microbiol.* 75 7537–7541. 10.1128/AEM.01541-09 19801464 PMC2786419

[B82] Slepova T. V. Sokolova T. G. Lysenko A. M. Tourova T. P. Kolganova T. V. Kamzolkina O. V. (2006). Carboxydocella sporoproducens sp. nov., a novel anaerobic CO-utilizing/H2-producing thermophilic bacterium from a kamchatka hot spring. *Int. J. Syst. Evol. Microbiol.* 56 797–800. 10.1099/ijs.0.63961-0 16585697

[B83] Smii L. Ben Hania W. Cayol J.-L. Joseph M. Hamdi M. Ollivier B. (2015). *Fusibacter bizertensis* sp. nov., isolated from a corroded kerosene storage tank. *Int. J. Syst. Evol. Microbiol.* 65 117–121. 10.1099/ijs.0.066183-0 25294821

[B84] Sokolova T. G. Kostrikina N. A. Chernyh N. A. Tourova T. P. Kolganova T. V. Bonch-Osmolovskaya E. A. (2002). Carboxydocella thermautotrophica gen. nov., sp. nov., a novel anaerobic, CO-utilizing thermophile from a kamchatkan hot spring. *Int. J. Syst. Evol. Microbiol.* 52 1961–1967. 10.1099/00207713-52-6-1961 12508854

[B85] Stringfellow W. T. Domen J. K. Camarillo M. K. Sandelin W. L. Borglin S. (2014). Physical, chemical, and biological characteristics of compounds used in hydraulic fracturing. *J. Hazard. Mater.* 275 37–54. 10.1016/j.jhazmat.2014.04.040 24853136

[B86] Strong L. C. Gould T. Kasinkas L. Sadowsky M. J. Aksan A. Wackett L. P. (2013). Biodegradation in waters from hydraulic fracturing: chemistry, microbiology, and engineering. *J. Environ. Eng.* 140:B4013001 10.1061/(ASCE)EE.1943-7870.0000792

[B87] Struchtemeyer C. G. Davis J. P. Elshahed M. S. (2011). Influence of the drilling mud formulation process on the bacterial communities in thermogenic natural gas wells of the barnett shale. *Appl. Environ. Microbiol.* 77 4744–4753. 10.1128/AEM.00233-11 21602366 PMC3147393

[B88] Struchtemeyer C. G. Elshahed M. S. (2012). Bacterial communities associated with hydraulic fracturing fluids in thermogenic natural gas wells in north central texas. *U S A. FEMS Microbiol. Ecol.* 81 13–25. 10.1111/j.1574-6941.2011.01196.x 22066833

[B89] Struchtemeyer C. G. Morrison M. D. Elshahed M. S. (2012). A critical assessment of the efficacy of biocides used during the hydraulic fracturing process in shale natural gas wells. *Int. Biodeterior. Biodegr.* 71 15–21. 10.1016/j.ibiod.2012.01.013

[B90] Waldron P. J. Petsch S. T. Martini A. M. Nüsslein K. Nüslein K. (2007). Salinity constraints on subsurface archaeal diversity and methanogenesis in sedimentary rock rich in organic matter. *Appl. Environ. Microbiol.* 73 4171–4179. 10.1128/AEM.02810-06 17468287 PMC1932797

[B91] Warner N. R. Jackson R. B. Darrah T. H. Osborn S. G. Down A. Zhao K. (2012). Geochemical evidence for possible natural migration of marcellus Formation brine to shallow aquifers in pennsylvania. *Proc. Natl. Acad. Sci. U. S. A.* 109 11961–11966. 10.1073/pnas.1121181109 22778445 PMC3409753

[B92] Winkel M. Pjevac P. Kleiner M. Littmann S. Meyerdierks A. Amann R. (2014). Identification and activity of acetate-assimilating bacteria in diffuse fluids venting from two deep-sea hydrothermal systems. *FEMS Microbiol. Ecol.* 90 731–746. 10.1111/1574-6941.12429 25244359

