# Supplementary material for: Microbial Metabolism and Community Dynamics in Hydraulic Fracturing Fluids Recovered From Deep Hydrocarbon-Rich Shale
Source: Front Microbiol. 2019 Mar 12;10:376. doi: 10.3389/fmicb.2019.00376 (PMC6422894; doi:10.3389/fmicb.2019.00376)
Supplement: Supplementary file 1 [file Data_Sheet_1.docx]

# Supplemental Tables and Figures

Supplemental Table 1 - Summary of contaminants that were removed from the 16S Illumina sequencing data.


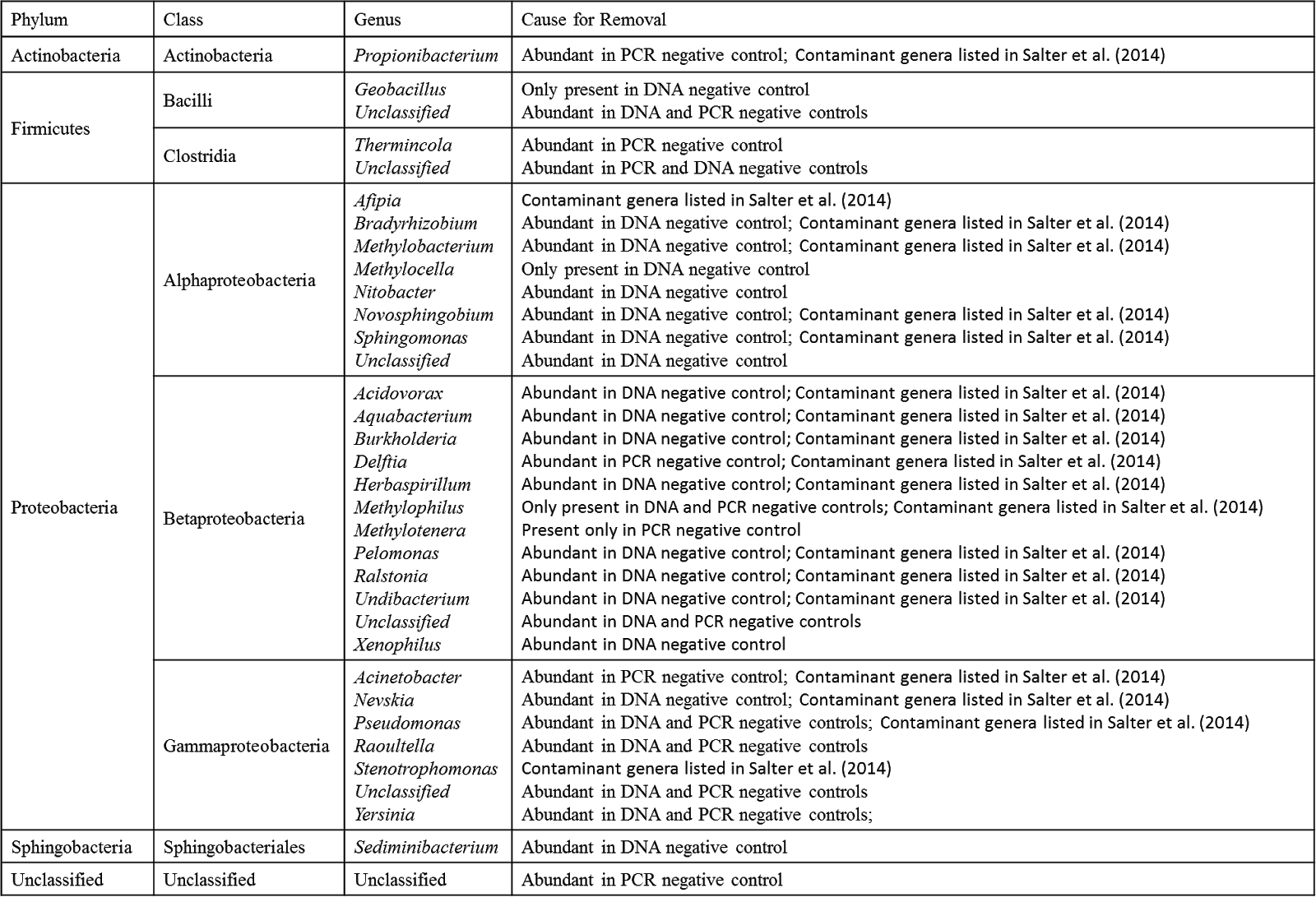


Supplemental Table 2 – Sequence numbers before and after contaminant removal for individual samples, as well as the number and percent of contaminant sequences that were removed.


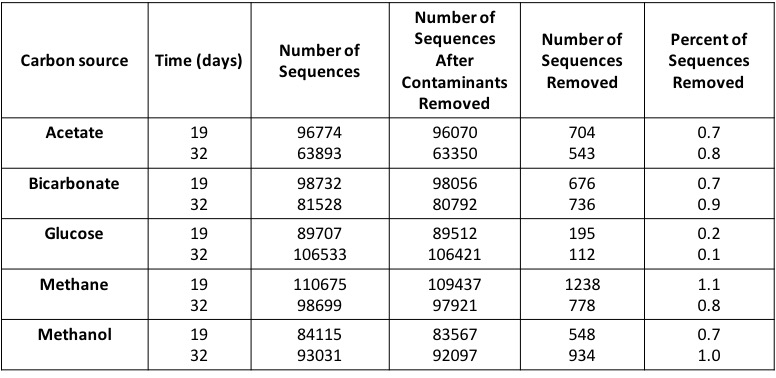


Supplemental Figure 1 - NanoSIMS images of microbial cells before incubation.  The left panel illustrates ^12^C^14^N ion counts that identify microbial cells.  The middle and right panel show the ratio image of ^13^C/^12^C and ^15^N/^14^N, respectively, showing no detectable increase in ^13^C/^12^C or ^15^N/^14^N observed for analyzed microbial cells.


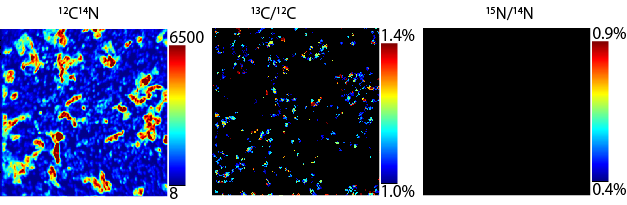


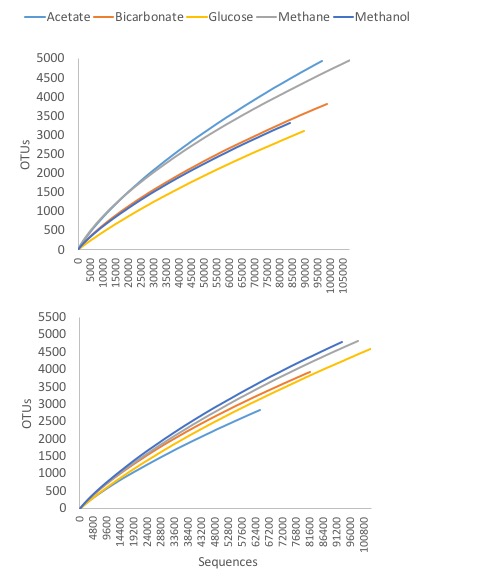
Supplemental Figure 2 - Rarefaction curves for flowback microbial communities at 19 (top), and 32 (bottom) days.
